# Pyrolysis of tea and coffee wastes: effect of physicochemical properties on kinetic and thermodynamic characteristics

**DOI:** 10.1007/s10973-022-11878-4

**Published:** 2023-02-10

**Authors:** Asma Ben Abdallah, Aïda Ben Hassen Trabelsi, María Victoria Navarro, Alberto Veses, Tomás García, Daoued Mihoubi

**Affiliations:** 1 Department of Energy Engineering, National School of Engineers of Monastir, University of Monastir, 5000 Monastir, Tunisia; 2 Laboratory of Wind Energy Management and Waste Energy Recovery (LMEEVED), Research and Technology Center of Energy (CRTEn), B.P. 95, 2050 Hammam-Lif, Tunisia; 3 Instituto de Carboquímica (ICB-CSIC), C/ Miguel Luesma Castán 4, 50018 Zaragoza, Spain

**Keywords:** Tea waste, Coffee waste, Pyrolysis, Kinetics, Thermodynamics

## Abstract

Physicochemical properties, kinetic pyrolysis and thermodynamic study of spent green tea, pure spent coffee grounds, spent coffee grounds blended with 50% torrefied barley and coffee husk were experimentally investigated using thermogravimetric analysis under an inert atmosphere to evaluate their thermochemical application. Five isoconversional methods were applied to determine effective activation energy (*E*_a_) of the pyrolysis processes. All methods showed good agreement by determining fluctuating *E*_a_ values (150–500 kJ mol^−1^). Complex *E*_a_ profiles with conversion were divided into four stages corresponding to thermal degradation of main biomass constituents (extractives, hemicellulose, cellulose and lignin), indicating that extractives decomposition was the least demanding reaction while lignin decomposition was the most demanding. The kinetic process was verified by reconstruction according to the Friedman parameters. The thermodynamic parameters were evaluated to determine the energy demand and efficiency throughout the process. The values obtained for physicochemical properties such as volatile matter (> 68%) and higher heating value (> 17 MJ kg^−1^), average *E*_a_ (223–319 kJ mol^−1^) and significant energy efficiency implied that these types of biomass waste have significant reactivity and consequently the highest potential for the production of bioenergy and a range of high-value chemicals and materials.

**Supplementary Information:**

The online version contains supplementary material available at 10.1007/s10973-022-11878-4.

## Introduction

In recent decades, the temperature on Earth has increased to values higher than those expected for 2100. As a result, soaring temperatures have led to harsher weather and the outbreak of fires, an example being the worst bushfires ever recorded in Australia that took place in December 2019. One of the main negative consequences of these issues is the continuously rising level of carbon dioxide (CO_2_) in the atmosphere. Although the carbon emissions declined by 5% in 2020 owing to the Coronavirus (COVID-19) pandemic and the economic shutdown, it ought to reduce 7.6% every year until 2030 to meet 1.5 ºC Paris target [1]. The use of renewable energy has become increasingly urgent as it can contribute to a reduction in our atmospheric carbon footprint while able to provide an inexhaustible source of clean energy.

Biomass is the most abundant, inexpensive and renewable organic source of energy and its appeal has aroused increasing interest in recent years. Biomass is expected to represent about 55% of the total renewable energy generated in the world by 2030 [2]. Examples of the most available waste biomass in the world, notably in Tunisia, are spent green tea leaves, spent coffee grounds and coffee husk. In 2020, world tea consumption reached 6.30 million tons and is estimated to reach to 7.4 million tons by 2025 [3]. Global coffee production in 2017–2018 was 9.5 million tons, increasing to around 10.2 million tons in 2018–2019 [4]. During the roasting step of green coffee beans to develop the characteristic flavor and taste of coffee, coffee husk is generated as by-product. In addition, consumption of coffee in beverage form generates an insoluble waste that accounts for 65% of its raw mass[5], whereas the waste generated by the consumption of tea accounts for 90% of its mass [6]. Part of the spent coffee waste is used as animal feed and for composting, although most of it is incinerated [7]. There are currently no applications for tea waste, which is disposed of in landfills.

Lignocellulosic biomass can be converted into energy through various processes, in particular thermochemical processes such as combustion, gasification and pyrolysis. Although it has a long history of use, pyrolysis has emerged as a frontier domain of research due to its efficiency in converting biomass to energy at a reasonable cost. It can produce liquid, solid and gaseous biofuels in a relatively simple stage in the direct thermal cracking of the biomass organic matrix in the absence of oxygen, typically at a temperature range between 400 ºC and 700 °C. However, it is a complex gas–solid thermochemical process that comprises heat and mass transfer to and from the particle and chemical reactions within the particle [8]. Besides, pyrolysis is subjected to the influences of various factors such as properties of biomass, heating rates, reactor patterns and conditions [9]. Therefore, it is highly relevant to acquire fundamental information on the biomass and the kinetics and thermodynamic properties of the pyrolysis reaction that are essential for engineering tasks such as designing and scaling up of industrial reactors, modelling, control and optimization of the process [8]. To this end, the thermal decomposition of different types of biomass has been studied with the data derived from thermogravimetric analysis (TGA). There are two classes of kinetic analysis methods: model-fitting methods and isoconversional methods. In recent years, more specifically after the developments of the International Confederation for Thermal Analysis and Calorimetry (ICTAC) Kinetics Committee [10], isoconversional methods have been increasingly utilized to investigate the kinetics of thermal decomposition of many types of biomass (e.g., cattle manure [11] and sour cherry stalk [12]) and/or their constituents [13]. Isoconversional methods allow the activation energy to be estimated as function of process temperature and heating rate for the whole reaction without prior knowledge of the reaction mechanism model. The basic hypothesis of these models is that the reaction rate is only a function of temperature at constant extent of conversion [10]. The isoconversional principle sets the basis for a large number of isoconversional computational methods, which can usually be split into two categories: differential (e.g., the Friedman method) and integral (e.g., the Kissinger–Akahira–Sunose (KAS), the Flynn–Wall–Ozawa (FWO), the Starink and the Vyazovkin methods).

In addition to the computation of kinetic parameters, the determination of thermodynamic data is highly significant when producing energy calculations and more particularly, in order to prove the feasibility of the process. Since the main objective of biomass pyrolysis process is the energy benefit, knowledge of the dependence of thermodynamic parameters (change of enthalpy (*ΔH*), entropy (*ΔS*) and Gibbs free energy (*ΔG*)) on process conditions is of vital importance. Because of the complexity and variability of biomass, the dependence of thermodynamic parameters on biomass properties such as proximate analysis, ultimate analysis and lignocellulosic constituents is one of the most important and challenge issues for the simulation and optimization of pyrolysis process of each kind of biomass [14].

Despite the existence of numerous studies on pyrolysis kinetics of biomass [15–17], to the best of our knowledge, limited studies are available in the literature on the kinetics of spent green tea, pure spent coffee grounds and coffee husk. Just recently, a study on thermokinetics of spent coffee grounds has been published relating activation energy with conversion and temperature [18]. Amezcua-Allieri et al. have recently published as well a study on the thermokinetic analysis of coffee husk [19]. The study shows that the degradation path of its main constituent at the maximum degradation rate responds to a first-order reaction. However, these studies do not provide the thermodynamic analysis of tea or coffee wastes pyrolysis neither the comparison with several samples, which is pivotal to ascertain he feasibility of the pyrolysis process and to compute energy needs. Moreover, no works have been found on the pyrolysis of spent coffee grounds blended with 50% torrefied barley. In addition, the composition, the chemical nature and quality of the residues differ depending on the type [20] and conditions for growing coffee beans and tea leaves and the type and number of process steps required to produce the beverages consumed in each country. Therefore, the study of the samples provided by Tunisian factories and coffee shops is of great interest.

The aim of this current research is to investigate both the thermal behavior and the pyrolysis kinetics of spent green tea and three types of coffee wastes by using thermogravimetric analysis performed in a non-oxidizing atmosphere. Activation energy was estimated by KAS, FWO, Starink, Vyazovkin and Friedman isoconversional methods. Furthermore, the pre-exponential factor and thermodynamic parameters (*ΔH*, *ΔG* and *ΔS*) were evaluated. The novelty of this research relies on establishing for the first time in the literature a relationship between the activation energy profiles and the biomass constituents (extractives, hemicellulose, cellulose and lignin) for a set of samples. For this, the four different samples were evaluated, providing valuable data about the influence of biomass constituents on the thermal degradation, kinetic and thermodynamic parameters. In addition, it is the first time in the literature that it is carried out the kinetic and thermodynamic analysis of spent coffee grounds blended with 50% torrefied barley. As a result, this research provides a significant basis for the valorization of these Tunisian wastes to support the transition to the circular economy of coffee industries and shops.

## Materials and methods

### Physicochemical characterization of biomass

The lignocellulosic biomass used in the current research were spent green tea (SGT), pure spent coffee ground (pure SCG) and SCG blended with 50% of torrefied barley (blended SCG) collected from a number of different coffee shops and coffee husk (CH) provided by a coffee factory. The fresh raw materials were naturally air-dried for 24 h before being used for pyrolysis process, except CH, which was obtained dried from the factory. The four dried samples were stored in a hermetic container for the different analyses. Proximate analyses of the four as-received samples were carried out according to the UNE-EN ISO 18134, UNE-EN ISO 18122 and UNE-EN ISO 18123 standards for moisture, ash contents and volatile matter (VM), respectively. Fixed carbon (FC) was calculated by balance. Ultimate analyses (CHNS) of the received wastes were performed using Thermo flash 1112 (UNE-EN 5104), while oxygen content was calculated by difference. The higher heating value (HHV, MJ kg^−1^) of the different feeds is determined using Eq. (1) reported by Chaniwala and Parikh’s correlation [21]:

1 $${\text{HHV}} = 0.{3491}\left( {\text{C}} \right) + {1}.{1783}\left( {\text{H}} \right) + 0.0{1}00{5}\left( {\text{S}} \right) - 0.{1}0{34}\left( {\text{O}} \right) - 0.0{151}\left( {\text{N}} \right) - 0.0{211}\left( {{\text{Ash}}} \right)$$

The analysis of four biomass constituents, namely extractives, hemicellulose, cellulose and lignin of all the studied lignocellulosic samples, was performed by deconvolution of the derivative thermogravimetric curves at 10 °C min^−1^ of the biomass using the peak fitting tool of OriginPro 2016 peak-fitting software [22].

### Thermogravimetric analysis

Thermogravimetric analysis (TGA) was performed in a Netzsch Libra F1 Thermobalance with a sensitivity of 0.0001 mg to provide different thermal decomposition patterns. In all cases, the sample mass and the particle size were fixed at 10 mg and 177–250 μm, respectively. The thermobalance was purged with N_2_ until stable mass was reached before starting the heating program. The powdered samples of the four types of lignocellulosic biomass were heated from room temperature to 900 °C using four different heating rates (10, 25, 50 and 100 °C min^−1^) to elucidate the different pyrolysis behaviors of those samples. The purge gas used during the thermogravimetric experiments was N_2_ at 50 mL min^−1^ in order to avoid the unwanted oxidation of different samples. The derivative thermogravimetry (DTG) curves for each sample were deconvoluted in order to determine the amounts of different constituents extractives, hemicelluloses, cellulose and lignin [22]. The deconvolution was based on the application of a Gaussian-type signal. The statistical measure (*R*^*2*^) value was 0.99 for the four deconvolutions indicating a good fit of the regression lines. All the decomposition data obtained from the TG analysis were used to assess kinetic and thermodynamic parameters.

The TG and DTG curves obtained through the measurement of the physical property of mass change related to temperature and time increase, it is a phenomenological approach that allows to describe the thermokinetic behavior of the samples. TG and DTG curves enabled also the identification of some parameters reflecting the characteristics of pyrolysis of different biomass wastes at different heating rates such as the initial and final temperature of thermal decomposition (*T*_i_ and *T*_f_, respectively), maximum mass loss rate (− *R*_p_), and its corresponding temperature (*T*_m_), average mass loss rate (− *R*_v_) and mass of pyrolysis residue (MR). In addition, a devolatilization index (*D*) was determined to quantify the characteristics of volatiles release, which is described according to Eq. (2) [23]:

2 $$ D = \frac{{ - R_{{\text{v}}} \cdot - R_{{\text{p}}} }}{{T_{{\text{i}}} \cdot T_{{\text{m}}}\cdot\Delta T_{1/2} }} $$

where $\Delta t_{1/2}$ defines the time zone of ${\text{ DTG}}_{{{\text{max}}}} = 0.5$.

### Kinetic study

The thermobalance records the variation in mass of the different samples during the process of thermal decomposition. Mass loss data were converted into degree of conversion, α, which is defined by $\alpha = \left( {m_{{\text{i}}} - m_{{\text{t}}} } \right)/\left( {m_{{\text{i}}} - m_{{\text{f}}} } \right)$ where *m*_i_, *m*_t_ and *m*_f_ are the initial sample mass, mass of sample at time t and final mass of sample, respectively. The expression of conversion rate was determined using the generalized form of the kinetic equation:

3 $$\frac{{{\text{d}}\alpha }}{{{\text{d}}t}} = k\left( T \right)f\left( \alpha \right)$$

where f (α) represents the conversion function and *k(T)* is the temperature dependence of the reaction rate constant as expressed by the Arrhenius equation.

Under non-isothermal conditions, temperature (*T*) and reaction time (*t*) were related as $T = T_{0} + \beta t$ (where *T*_0_ and *β* represent the initial temperature of reaction and heating rate, respectively). At a constant heating rate, Eq. (3) is converted into a derivative form of temperature:

4 $$\beta \frac{{{\text{d}}\alpha }}{{{\text{d}}T}} = A\exp \left( {\frac{{ - E_{{\text{a}}} }}{RT}} \right)f\left( \alpha \right)$$

where *A*, *R* and *E*_a_ represent the pre-exponential factor, universal gas constant (8.314 J mol^−1^ K^−1^) and activation energy, respectively.

Isoconversional methods are recommended by the ICTAC [10] for the kinetic analysis of complex solid-state thermochemical reactions and to provide effective *E*_a_. According to Wu et al. [24], effective *E*_a_ is a composite value determined by the *E*_a_ of simultaneous processes. Four integral isoconversional methods (KAS, FWO, Starink and Vyazovkin methods) and the isoconversional Friedman method in the differential form [10] were applied in this work to calculate *E*_a_. Table S1 shows the mathematical expressions of these methods. These isoconversional methods enabled estimation of *E*_a_ at different conversion values, avoiding the uncertainty introduced by the reaction model assumption.

### Verification of kinetics

In most cases, isoconversional methods result in only activation energies being estimated, which cannot lead to reproducing data of reaction rate. The calculation of the involved pre-exponential factor needs to assume a kinetic model and the application of isoconversional method is no longer model free. As a result, the calculated *E*_a_ may or may not be well fitting the experimental data. However, based on the values of *E*_a_ and ln[*A*f(α)] obtained from Friedman method and according to Eq. (4), the following equation is obtained to reconstruct the kinetic process [25]:

5 $$\frac{{{\text{d}}T}}{{{\text{d}}\alpha }} = \beta \exp \left( {\frac{{E_{{\text{a}}} }}{RT} - \ln \left[ {Af\left( \alpha \right)} \right]} \right)$$

In order to assess the reproduction of experimental data, the often used *R*^*2*^ statistic was used in addition to the mean absolute error (MAE) and root-mean-square error (RMSE), which are expressed through the following equations [26]:

6 $${\text{MAE}} = \frac{1}{N}\mathop \sum \limits_{k = 1}^{N} \left| {\left( {\beta \frac{{{\text{d}}T}}{{{\text{d}}\alpha }}} \right)_{{\text{k,exp}}} - \left( {\beta \frac{{{\text{d}}T}}{{{\text{d}}\alpha }}} \right)_{{\text{k,sim}}} } \right|$$

7 $${\text{RMSE}} = \sqrt {\frac{{\mathop \sum \nolimits_{k = 1}^{N} \left[ {\left( {\beta \frac{{{\text{d}}T}}{{{\text{d}}\alpha }}} \right)_{{\text{k}},\exp } - \left( {\beta \frac{{{\text{d}}T}}{{{\text{d}}\alpha }}} \right)_{{\text{k,sim}}} } \right]^{2} }}{N}}$$

where N represents the number of used experimental points, and the subscripts exp and sim denote the experimental and the simulated data, respectively.

### Pre-exponential factor and thermodynamic analysis

During this study, pre-exponential factor (*A*) and the thermodynamic parameters of the different samples including *ΔH*, *ΔS* and *ΔG* were evaluated. Since *E*_a_ values calculated applying Friedman method were selected to follow the study, *A* was calculated from the previous defined Langmuir parameter ln*[Af(α)]* assuming a kinetic model for the reaction of first order [27]. The following equations [28] can express the thermodynamic parameters related to the activated complex of chemical reactions [29] by using the values of *E*_a_ obtained from Friedman method:

8 $$\Delta H = E_{\upalpha } - RT_{\upalpha }$$

9 $$\Delta G = E_{\upalpha } + R \cdot T_{{\text{m}}} \cdot \ln ((K_{{\text{B}}} \cdot T_{{\text{m}}} )/\left( {h \cdot A} \right))$$

10 $$\Delta S = \left( {\Delta H - \Delta G} \right)/T_{{\text{m}}}$$

where *K*_B_ is the Boltzmann constant (1.381 × 10^–23^ J K^−1^), h is the Plank constant (6.626 × 10^–34^ J s) and *T*_m_ is the DTG peak temperature.

## Results and discussion

### Physicochemical properties of raw materials

The physicochemical characteristics of SGT, pure SCG, blended SGC and CH are shown in Table 1. Before starting the pyrolysis process, the proximate analysis of biomass provides data about their potential use as raw material. Table 1 shows that moisture content is lower than 10% in all the types of biomass waste, which can favor the production of bioenergy by the pyrolysis process [30]. Particularly, pure SCG has the highest moisture content value and SGT the lowest (9.29% and 7.24%, respectively), despite undergoing the same steps during drying. This result could be related to the presence of moisture remaining in the cellulose of pure SCG. The moisture content in feedstock affects the properties of pyrolysis products. In fact, a higher moisture content leads to lower heating value of the liquid fraction having a lower heating value. Ash represents the undesirable inorganic fraction in the feedstock that remains after the combustion step. The lowest ash content was found in pure SCG, while the highest was in CH (see Table 1). These values are comparable to the ash content of different types of lignocellulosic biomass, such as corn husk [31] and poplar fluff [17]. Interestingly, low ash content results in lower level of aggregation or fouling in combustors, while high ash content causes problems with disposal, has disadvantageous effects on processing and leads to lower efficiency in the energy generation. Volatile matter and fixed carbon values represent the stored energy in the feedstocks. Table 1 shows that the fixed carbon of the four types of biomass waste varied in the following order: blended SCG > SGT > CH > pure SCG. Volatile matter varied between 68.58 to and 73.91% increasing in the following order: CH < blended SCG < SGT < pure SCG. The VM/FC ratio is an indicator of the degradability of biomass through the initial conversion phase [32]. Thus, the higher the VM/FC ratio, the higher the availability of energy in the raw materials. Ultimate analysis is useful to compute the calorific value of the biomass and to indicate its fuel efficiency. The carbon content of all samples, which was in the range of 45–51%, showed that the studied lignocellulosic samples could be feasible for the production of biofuel. This organic element was found to be similar to other lignocellulosic biomasses [15, 33, 34]. The nitrogen and sulfur contents of the feedstocks were in the range of 2.31–2.92% and 0.08–0.18%, respectively. Interestingly, contents in these elements were lower than those found in certain lignocellulosic biomass wastes that were already treated via pyrolysis such as Eucalyptus leaves and another type of coffee husk [27, 35]. Biomass types with low nitrogen and sulfur contents are very attractive for the pyrolysis process since further combustion of obtained biofuels will lead to low emissions. The HHV for all the studied samples were found to be in the range of 17.79–21.52 MJ kg^−1^. The HHVs of tea and coffee wastes are higher when compared to the calorific values of other types of lignocellulosic biomass such as poplar fluff (15.86 MJ kg^−1^) [17], grass (15.04 MJ kg^−1^) [36] and rice husk (12.9 MJ kg^−1^) [27]. Therefore, the four studied feedstocks show great potential for as feedstocks for the production of clean energy to replace solid fossil fuels.

**Table 1:** Feedstock properties

	SGT	Pure SCG	Blended SCG	CH
*Proximate analysis/%*				
Moisture	7.24	9.29	8.19	7.30
Ash	3.75	1.66	2.00	8.03
VM	70.25	73.91	70.22	68.58
FC^a^	18.76	15.14	19.59	16.09
VM/FC	3.75	4.88	3.58	4.26
*Ultimate analysis/%*				
C	46.12	48.69	51.05	45.04
H	6.47	6.89	6.68	5.96
N	2.79	2.31	2.61	2.92
S	0.12	0.08	0.08	0.18
O^a^	44.50	42.03	39.58	45.90
HHV/MJ kg^−1^	19.00	20.71	21.52	17.79

^a^By difference; C: Carbon; H: Hydrogen; N: Nitrogen; S: Sulfur; O: Oxygen

### Thermal degradation behavior

**Fig. 1:**
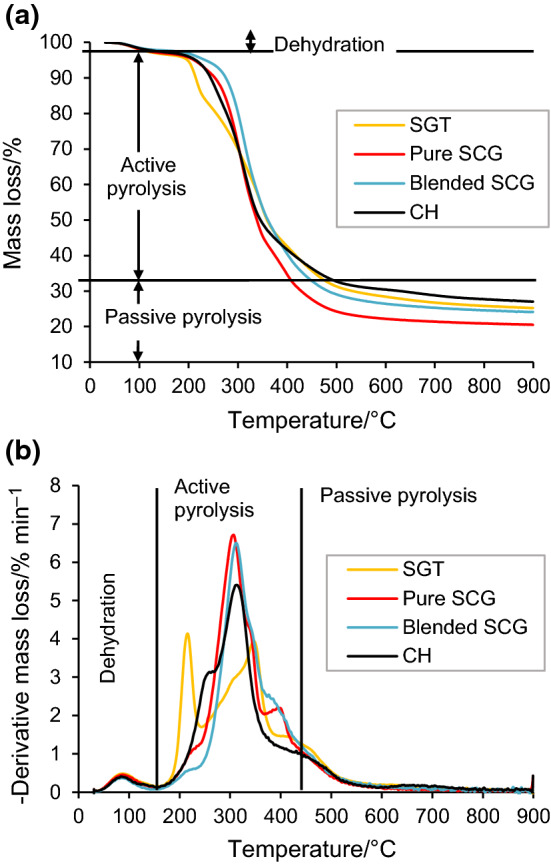
Comparison between (**a**) the TGA and (**b**) the DTG profiles of four samples at 10 °C min^−1^

The TGA and DTG curves of SGT, pure SCG, blended SCG and CH at 10 °C min^−1^ are illustrated in Fig. 1a, b. The thermal decomposition process of the four samples was divided into three pyrolytic stages (Fig. 1a). The first stage, which occurred within a temperature range of 30–180 °C, corresponded to the release of moisture (dehydration). During this pre-pyrolysis stage, the weakly bonded H_2_O was released owing to the hygroscopic nature of the biomass. The mass loss of the SGT, pure SCG, blended SCG and CH was 3.5%, 3.3%, 2.5% and 2.8%, respectively. The second stage, characterized by the largest mass loss, was recognized as the active pyrolysis stage. This stage took place at higher temperatures, 180–600 °C, and involved the higher molecular mass compounds being fragmented into various smaller molecules. During this stage, the devolatilization of extractives, hemicellulose, cellulose and lignin occurred, leading to the release of gases and different vapors. By virtue of the high volatile matter content and high degradability value (VM/FC ratio) of the pure SCG, the mass loss percentage of this sample (73.6%) was higher than those of the blended SCG (69.8%), SGT (66.4%) and CH (65.9%). The third stage is attributed to the passive pyrolysis that took place within the temperature range of 450–900 °C and corresponded to the endothermic thermal decomposition of the remaining lignin. Compared to the active pyrolytic stage, the degradation rate of all the samples at this stage was very low because of the recalcitrant nature of lignin, which indicated the absence of appreciable mass conversion reactions. The total mass loss of SGT, pure SCG, blended SCG and CH at this stage was 6.3%, 12.1%, 6.5% and 5.1% of the original mass, respectively. The highest mass loss of pure SCG within the passive pyrolysis zone could be explained by the high amount of lignin with a complex structure. As lignin consists of highly branched groups of C–H and phenylpropane molecules, this complex polymer is very difficult to degrade [27].

The DTG curves of the four samples (Fig. 1b) differ in the position and height of the peaks. These different patterns demonstrate that thermal decomposition behavior was affected by the different physicochemical properties of the residues and the distribution of the biomass constituents, namely extractives, hemicellulose, cellulose and lignin. By studying the active pyrolysis stage of the samples, three different peaks and a flat tailing section could be observed. The first shoulder/peak observed within the temperature range of 180–255 °C was linked to the loss of extractives. Two more DTG peaks corresponded to the thermal decomposition of hemicellulose and the cellulose taking place at 200–350 °C and 250–400 °C, respectively. The decomposition of this cellulosic part has been described as taking place in two steps: firstly, the formation of CO_2_, CO and hydrocarbons from the breakdown of polymers and molecular bonds (C–H and C–C bonds) at low temperatures; and secondly, at higher temperature, with the formation of condensable vapors resulting from the formation of bonds between released radicals [28]. Finally, the decomposition of certain amount of lignin is represented by the flat tailing section of the derivative curves in the last part of the active pyrolysis up to 430 °C. The lignin decomposed slowly over the very broad temperature range of 180–900 °C [22]. Obviously, the heating rate of any pyrolysis process is a parameter of paramount importance as this process variable not only influences both biomass conversion and the distribution of pyrolysis products but also determines the type of reactor that should be used in industrial processes [37].

**Fig. 2:**
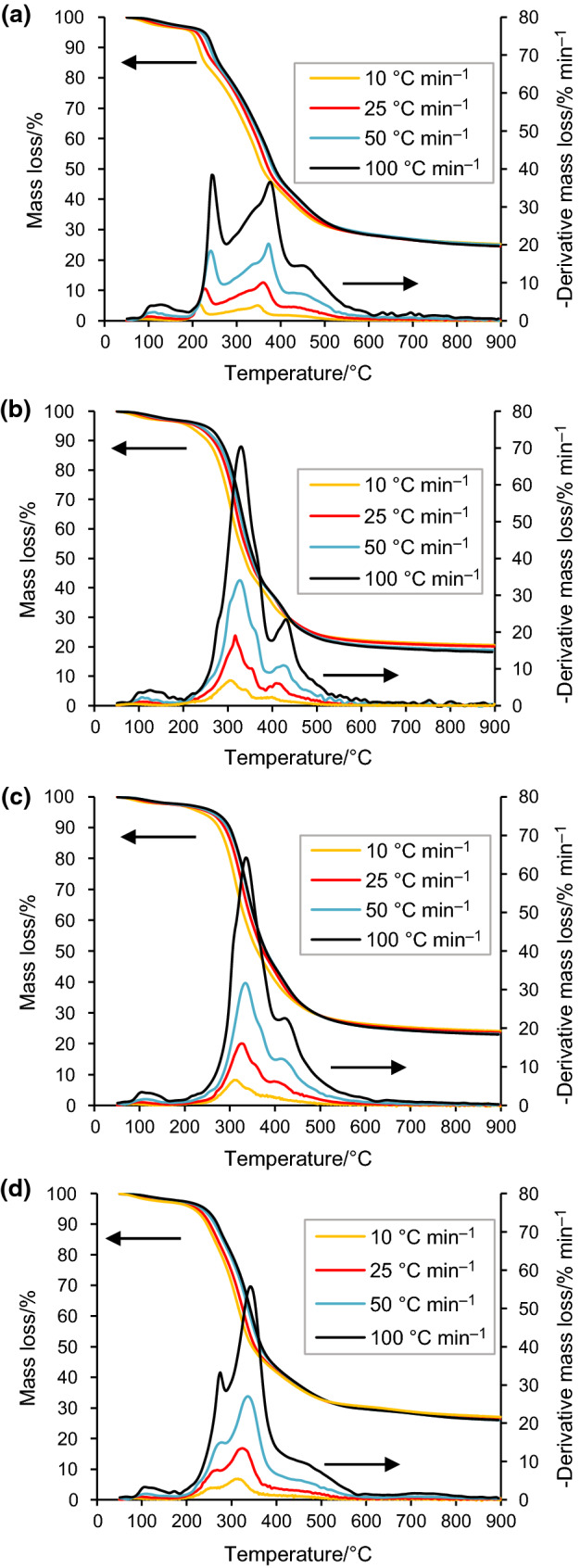
TG and DTG profiles of (**a**) SGT, (**b**) pure SCG, (**c**) blended SCG, and (**d**) CH at 10, 25, 50 and 100 °C min^−1^

Figure 2 and Table 2 clearly show that the heating rate significantly affected the thermal decomposition range and the characteristic parameters of pyrolysis for different biomass wastes, respectively. The residues percentage of the four samples, except SGT, was reduced by raising heating rates from 10 to 100 °C min^−1^. These results could be explained by the rapid decomposition of lignocellulosic components of samples at higher heating rates, resulting in high volatile yields and in reduction time of secondary coking reaction. The different characteristic temperatures of the pyrolysis process (*T*_i_, *T*_m_ and *T*_f_) also rose with the increasing heating rates. It can be observed from Fig. 2 that the increase in heating rates significantly enhanced the mass loss rate and shifted the peak temperature, and TG and DTG curves to higher values of temperature. As a result, -*R*_p_ and *D* values increased, proving that the higher heating rates may improve the pyrolysis process. From Table 2, it should be highlighted that *T*_i_ and *T*_f_ of the four samples are different, which could be attributed to the difference in the amounts of extractives, hemicellulose, cellulose and lignin as well as their inorganic contents. The -*R*_v_ value of CH was lower compared to that of SGT, and pure and blended SCG, indicating that the stability of CH pyrolysis was higher than the other wastes. Moreover, the ash content in CH was higher, which reveals more inorganic minerals, resulting in a great percentage of residue. The − *R*_v_ and *D* values of pure and blended SCG were significant, which implied that they had great devolatilization performance in comparison with CH and SGT. The higher the values of − *R*_v_ and *D*, the more reactive the pyrolysis reaction [38].

**Table 2:** Characteristic parameters of pyrolysis for the four biomass wastes at different heating rates

Fuels	*β*	*T*_i_/°C	*T*_m_/°C	*T*_f_/°C	− *R*_p_/% min^−1^	-*R*_v_/% min^−1^	MR/%	*D*/%^2^ °C^−3^ min^−2^
SGT	10	174.2	348.2	512.4	4.04	0.89	25.24	5.84 × 10^–7^
	25	181.7	360.6	542.3	10.07	2.23	24.51	2.51 × 10^–6^
	50	187.7	373.5	566.9	20.24	4.47	25.03	9.89 × 10^–6^
	100	191.9	374.1	612.6	38.35	9.05	24.58	2,87 × 10^–5^
Pure SCG	10	171.0	303.8	501.7	6.83	0.94	20.49	1,64 × 10^–6^
	25	183.7	316.7	511.7	19.03	2.36	20.08	1.11 × 10^–5^
	50	185.8	327.7	521.0	34.03	4.84	18.83	3.52 × 10^–5^
	100	204.3	330.8	544.9	70.32	9.81	18.20	1.33 × 10^–4^
Blended SCG	10	174.3	309.4	480.3	6.59	0.89	24.16	1.49 × 10^–6^
	25	186.7	326.6	512.3	16.05	2.26	23.62	7.97 × 10^–6^
	50	197.8	333.7	552.9	31.71	4.58	23.20	2.88 × 10^–5^
	100	204.3	334.5	588.8	64.15	9.24	23.01	1.11 × 10^–4^
CH	10	166.2	314.6	438.6	5.49	0.86	27.13	9.40 × 10^–7^
	25	169.7	323.7	488.9	13.44	2.17	26.45	5.22 × 10^–6^
	50	179.8	335.7	545.0	27.02	4.41	26.05	1.91 × 10^–5^
	100	183.8	342.5	580.8	55.73	8.87	26.04	8.05 × 10^–5^

DTG curves were used to determine the amounts of lignocellulosic constituents [22]. Resulting relative area contributions of the different biomass constituents to the total main DTG profiles are compiled in Table 3 with graphical representations shown in Fig. S1 (Supplementary Information). Based on these percentages, SGT contained the largest amounts of extractives and hemicellulose: 11.7% and 34.0%, respectively. On the other hand, the highest contents in cellulose (49.8%) and lignin (36.7%) were shown for pure SCG and blended SCG, respectively, whereas CH had the lowest lignin content (25.6%). These values are in line with those collected from the literature [42]. Nevertheless, a number of important factors, such as crop variety, cultivation conditions and processing methods, as well as the analytical method used to determine biomass composition, could explain the minor differences found, particularly with regard to the CH sample.

**Table 3:** Relative contribution of the four biomass constituents derived from deconvolution at 10 °C min^−1^/%

Biomass	Extractives	Hemicellulose	Cellulose	Lignin	References
SGT	11.7	34.0	21.5	32.7	This work
Pure SCG	3.0	15.4	49.8	31.9	This work
Blended SCG	3.9	10.5	48.9	36.7	This work
CH	3.4	26.7	44.4	25.6	This work
Tea waste	13.9	31.1	25.4	25.7	[39]
Spent coffee grounds	–	19.0	47.3	29.3	[5]
Coffee husk	6.7	25.5	26.5	33.5	[40]
Coffee husk	–	7.0	43.0	9.0	[41]

### Kinetic analysis

Biomass pyrolysis is a complex process. Therefore, in order to provide an efficient design for this thermochemical conversion, it is crucial to thoroughly understand pyrolysis kinetics. In the process of kinetic analysis, isoconversional KAS, FWO, Starink, Friedman and Vyazovkin methods were utilized to compute *E*_a_ for the four biomass samples. As this research focuses on the process of thermal decomposition, the chosen temperature range was around 180–600 °C, because the drying step was not taken into account during pyrolysis analysis and there was only negligible loss after 600 °C. In addition, the reaction temperature generally used in the most often performed process of fast pyrolysis of biomass to maximize bio-oil production is 500 °C [43] that falls in this range. Hu et al. [44] noted that under low or high conversions, errors could take place in the determination of data using the isoconversional methods with large fluctuations generated in the kinetic parameters. Accordingly, the conversion range selected for the kinetic analysis was 10–90% with a 5% step size to calculate the kinetic parameters for all samples.

The minimum and maximum correlation coefficients (*R*^*2*^) (Table S2, Supplementary Information) were determined for all samples and for each isoconversional method within the range of 0.93–1.0, which indicated the high accuracy and reasonability of the analysis methods used. In the Vyazovkin method, the computed percentage errors in *E*_a_ were lower than 4.85%.

**Fig. 3:**
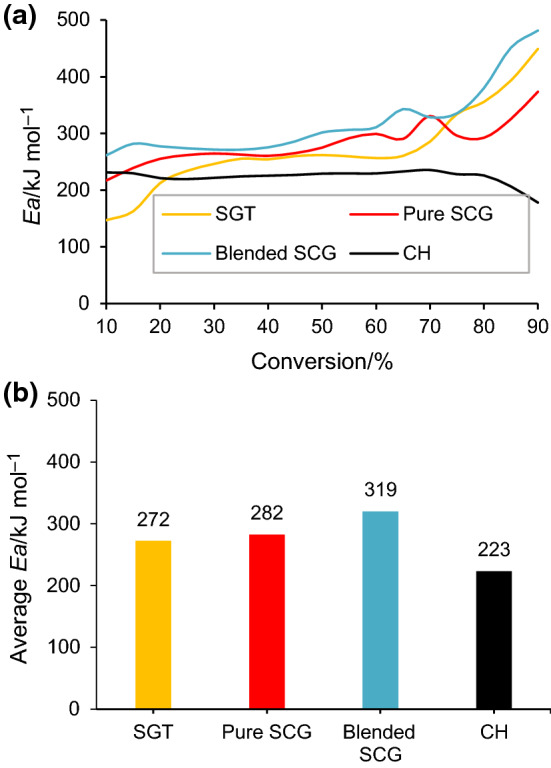
**(a)** *E*_a_ as a function of conversion and (**b)** average *E*_a_ from Friedman model

*E*_a_ values are influenced by different factors, such as type of biomass and kinetic models. The *E*_a_ obtained from the different isoconversional methods were relatively close to each other and their profiles showed the same trend with increased conversion for all lignocellulosic biomass wastes (Fig. S2, Supplementary Information). The results of *E*_a_ computed from the integral methods were practically superimposed. Similarly, Oliveira et al. [13] and Arenas et al. [15] observed quite comparable profiles of conversion–*E*_a_ using different integral isoconversional methods for energy cane and agro-industrial by-products, respectively. It should be noted that all these methods are based on the assumption of the temperature integral, assuming that *E*_a_ is constant at each conversion range. This approximation presents a source of systematic errors in *E*_a_ values as pointed out by Vyazovkin et al. [10]. On the other hand, the *E*_a_ profiles obtained by applying the differential Friedman isoconversional method are compiled in Fig. S2 (Supplementary Information), where some differences in the activation energy profile can be observed for each sample. Compared to integral methods, differential method does not involve any approximation for assessing the temperature function. Moreover, this differential method is not restricted to using a linear variation in the heating rate [25]. *E*_a_ profiles for the different samples using the Friedman method are compared in Fig. 3a. The values presented for CH are consistent with the values calculated by Amezcua-Alleri et al. [19] applying the Friedman model and taking into account an individual decomposition of the constituents, 215–242 kJ for hemicellulose and 213 kJ mol^−1^ for cellulose. Additionally, average *E*_a_ values are depicted in Fig. 3b. The order of this average value of the four samples was found to be CH < SGT < pure SCG < blended SCG. The average *E*_a_ values reported in this study were in the same range as the *E*_a_ of different feedstocks mentioned in literature, such as castor husk (215.6 kJ mol^−1^), palm kernel shell (262 kJ mol^−1^) and corn stalk (473 kJ mol^−1^) [27, 28]. The variation in *E*_a_ for the SGT and different coffee wastes should be linked to their differing VM and FC content and composition (extractives, hemicellulose, cellulose and lignin). Among the four feedstocks, blended SCG had the highest *E*_a_ value (as shown in Fig. 3b). This could be ascribed to its high lignin content and low VM/FC ratio, decreasing its reactivity. Conversely, CH was found to have the lowest activation energy among the different investigated fuels. Low *E*_a_ values could be linked to the highest (cellulose + hemicellulose) content observed for this sample, together with a significant VM/FC ratio. Additionally, it cannot be ruled out that a high ash content acting as catalyst could exert a positive role in the reactivity of the sample, as previously reported for other biomass samples [45], given that CH is the sample with the highest ash content and the lowest *E*_a_ [27, 45].

**Fig. 4:**
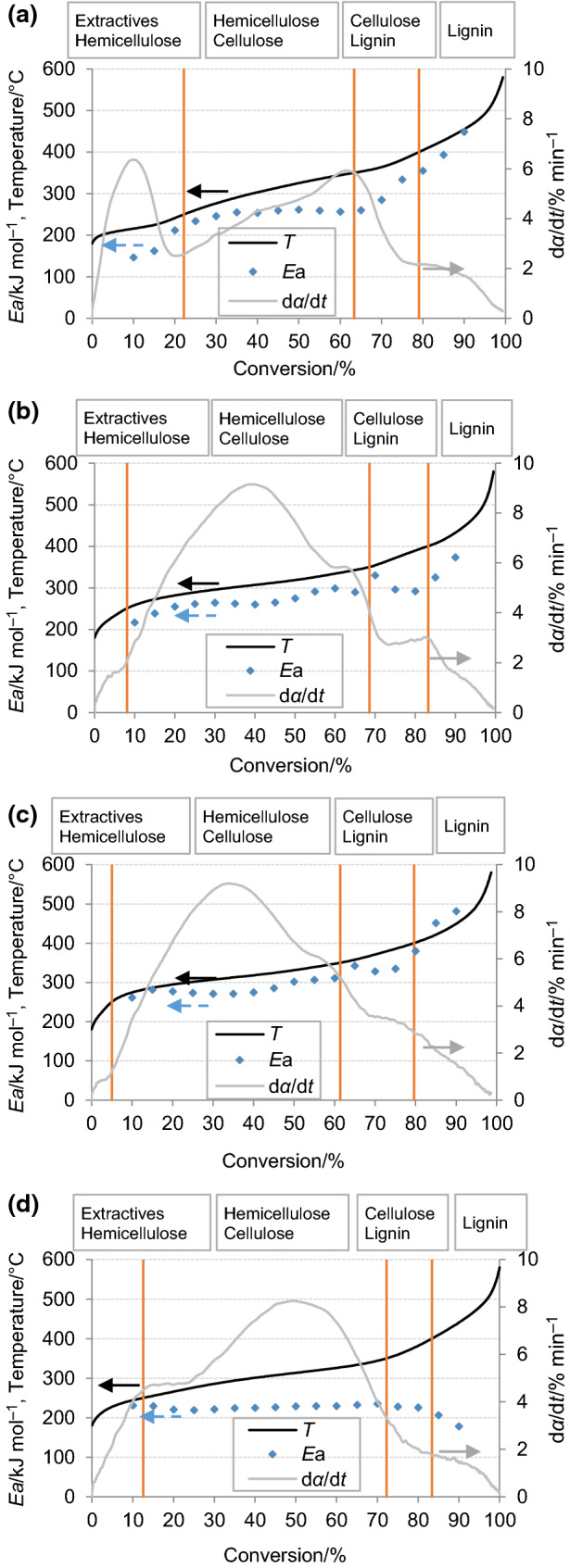
Temperature, reaction rate and *E*_a_ as a function of conversion and biomass constituents for **a** SGT, **b** pure SCG, **c** blended SCG and **d** CH at 10 °C min^−1^

The values of *E*_a_ obtained at different conversion levels exhibit some important fluctuations that suggest the appearance of various complex and multi-step reactions as the pyrolysis temperature is increased (including competitive, consecutive and parallel reactions) [10, 15]. The *E*_a_ profiles of the biomass waste (Fig. 4) were tentatively divided into four stages established based on temperatures ranges defined for the decomposition of the different biomass constituents [22]: (1) 180–255 °C, thermal degradation of extractives and some of the hemicellulose content; (2) 255–350 °C, decomposition of the main part of hemicellulose and cellulose; (3) 350–400 °C, devolatilization of lignin together with the remaining cellulose; and (4) > 400 °C, degradation of the remaining lignin during charring reactions. This division is supported on the assumption of an added pyrolysis behavior of biomass constituents that have been previously reported for a wide range of biomass samples [46].

The definition of these four stages in relation to conversion is different for each of the studied samples (see Fig. 4) because the conversion degrees changed when reaching the fitted temperatures since each sample had a different proportion of biomass constituents as previously calculated and compiled in Table 3. For instance, the first stage for SGT corresponded to more than 20% conversion compared to less than 10% for pure SCG and blended SCG, given that SGT had the highest content in extractives and hemicellulose. On the other hand, CH was characterized by the highest content in cellulose + hemicellulose and it therefore presented the broadest window for the second stage, around 65 percentage points. There are a number of observations to highlight with regard to the evolution of *E*_a_. In the first stage, at initial conversion percentages, the *E*_a_ of SGT was very low compared to the other samples, likely due to the highest content in extractives in this biomass waste since extractives enhance the reactivity of different constituents and improve the decomposition of various structural compounds [31]. The increase in the *E*_a_ observed from 15 to 22% of conversion reveals an alteration on the thermokinetic behavior at 250 °C where hemicellulose devolatilization seems to have been achieved. The breakdown of the weakly attached sites inherent to the linear chains of hemicellulose polymers followed by further random rupture of linear chains may have been taking place [47]. An *E*_a_ value of around 200 kJ mol^−1^ was obtained, which is in line with those found in the literature [46, 48]. Accordingly, for the CH sample, which had a lower content in extractives, this first decomposition stage with *E*_a_ values of around 200 kJ mol^−1^ corresponded directly to hemicellulose decomposition.

In the second stage, while hemicellulose decomposition was fully accomplished, the cellulose molecules start to devolatilize, leading to an increase in the *E*_a_. *E*_a_ values higher than 250 kJ mol^−1^ were obtained, which are in agreement with those observed in the literature for cellulose pyrolysis [46, 48]. SGT and CH show very close values of activation energy throughout the stage, meaning that the degradation occurs at energetically equivalent sites based on the presence of linearly ordered polymeric chains in cellulose [49]. For pure SCG and blended SCG, the variation of *E*_a_ in this stage is higher indicating the existence of some cooperative phenomena, which involve complex and competitive process typical of multi-step reactions related to the breaking of various bond types energetically different inside the polymer matrix [50, 51].

In the third stage, the decomposition of the remaining cellulose is completed, also starting to be lignin devolatilization relevant. Specifically, a high increase of *E*_a_ is observed in this stage for SGT sample. This phenomenon was previously linked to a different nature of the cellulose fibers, as reported by Kaur et al. [28]. For pure SCG and blended SCG, the *E*_a_ values increased slightly and then decreased. When the cellulose pyrolysis was completed, the interaction with lignin disappeared and *E*_a_ decreased to a constant value [47]. This stage is a multi-step process supported on the initial interaction of remaining cellulose with lignin and the later lignin degradation. It follows  a multi-component and multi-phase reaction system where the first values of *E*_a_ (340 kJ mol^−1^) reflect the existence of several pyrolytic derivatives (solid, liquid and gaseous) which impact decreases while their concentration decreases. The lower *E*_a_ value (300 kJ mol) is related to lignin degradation taking place in the presence of a mixture of by-products (i.e., tars, carbonaceous residues, ash and gases) [18]. For CH, degradation of the remaining cellulose was the main process taking place with energetically equivalent bonds [50] and the *E*_a_ value was mostly constant.

The fourth stage corresponded to the thermal degradation of the remaining lignin at high temperatures. A sharp increase in the *E*_a_ is shown for SGT, pure SCG and blended SCG (334–449 kJ mol^−1^, 292–374 kJ mol^−1^ and 336–482 kJ mol^−1^, respectively) with the rise in temperature. This behavior could be explained by the increase in the main lignin molecule decomposition [17]. At higher temperatures, the lignin-derived char content in the solid residue of these samples increases, which has high thermal stability and low reactivity. This lignin-derived char is formed by means of uninterrupted cross-linking and expansion of the aromatic compounds. When the number of active sites was reduced, the reaction activity decreased rapidly, leading to an important increase in the *E*_a_ [52]. Brachi et al. [18] observed a similar increase in *E*_a_ at high conversion percentages of SCG and described the lignin degradation taking place at this stage in the presence of a mixture of by-products through a multi-component and multi-phase reaction system. The increase of the concentration of pyrolytic derivatives (i.e., tar, carbonaceous residues, ash and gases) increases the value of *E*_a_. Similar behavior was found for sour cherry stalk and flesh at high conversion rates [12]. Within the same stage, the *E*_a_ value for CH decreased constantly from 226 kJ mol^−1^ to 178 kJ mol^−1^ with increasing conversion, indicating that the endothermic reaction was followed by an irreversible step (exothermic reaction) [53]. These results contrast with the result obtained by Amezcua-Alleri et al. [19] applying the Friedman model with an increase in the *E*_a_ value for lignin up to 280.7 kJ mol^−1^.

Taking into account the variance in the physicochemical properties of the four tested samples and operating conditions, the results obtained in this study exhibited similar trends to some other results reported in the literature [11, 18, 30]. Specifically, Brachi et al. [18] recently studied the pyrolytic degradation of SCG and calculated the profile of *E*_a_ versus conversion applying the Friedman model to obtain a very similar trend and values (240 to 400 kJ mol^−1^). The authors performed as well an analysis on the influence of constituents (hemicellulose, cellulose and lignin) of the SCG sample on the *E*_a_ profile to establish four differentiated stages of degradation slightly different to this work. For the SCG, after a first initialization stage, they take into account the constituents mainly degrading in each stage with some overlapping between hemicellulose and cellulose in the third stage.

### Verification

**Fig.5:**
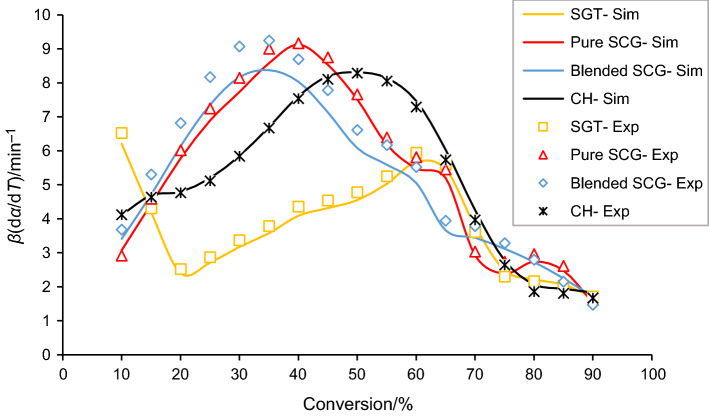
Comparison between the experimental (Exp) and simulated (Sim) data for SGT, pure SCG, blended SCG and CH pyrolysis at 10 °C min^−1^

Figure 5 depicts the comparison between experimental and simulated data for the four biomass wastes at 10 °C min^−1^. It is noticed that kinetic simulations were well-matched with experimental data, indicating the effectiveness of resulted kinetic parameters. Moreover, the ability of the parameters obtained to reproduce the kinetic process was evaluated through the comparison of simulated and experimental data in terms of MAE, RMSE and *R*^*2*^, listed in Table S3 (Supplementary Information). Based on *R*^2^ values, all the simulations show a very good agreement with values higher than 0.96. Regarding the values obtained of MAE and RMSE, some differences can be observed regarding the heating rate. In fact, the most accurate simulation results were obtained at the lowest heating rate for all the samples due to the lower values of the kinetic parameter compared to higher heating rates. However, all the values obtained are comparable to values obtained previously in the literature for this parameter [26].

### Pre-exponential factor and thermodynamic study

For a good understanding of the biomass pyrolysis process, it is essential to determine the pre-exponential factor (*A*) and the thermodynamic parameters such as change of enthalpy (*ΔH*), change of Gibbs free energy (*ΔG*) and entropy (*ΔS*). The estimation of the three thermodynamic parameters defines the measurement of energy needs and the energy efficiency of the biomass pyrolysis process. Based on the *E*_a_ values obtained through the Friedman method (illustrated in Fig. 3a), the values of the thermodynamic parameters for the four biomass samples were calculated at different heating rates (10, 25, 50 and 100 °C min^−1^).

In general, *A* reflects the complexity of the reaction or sample surface structure during the pyrolysis process [38]. The *A* values, compiled in Table S4 (Supplementary Information), show variations in a wide range between 5.4 × 10^12^ and 4.7 × 10^29^ s^−1^ for SGT, between 1.1 × 10^18^ and 1.1 × 10^25^ s^−1^ for pure SCG, between 5.4 × 10^21^ and 1.9 × 10^32^ s^−1^ for blended SCG and between 3.2 × 10^10^ and 1.2 × 10^20^ s^−1^ for CH. According to Vuppaladadiyam et al. [54], pre-exponential values higher than 10^14^ s^−1^ indicate more difficult and slower degradation processes because the solid stores energy (potential energy), increasing the frequency of vibration in the molecules and atoms that compose it, until reaching a critical energy state, where the stored potential energy is transformed into kinetic energy [18]. Table S4 (Supplementary Information) shows high A values that suggest a more difficult pyrolysis process. The fluctuations in *A* with conversion imply that the composition of the four types of biomass waste was complex and the pyrolysis reactions at different α were complex as well. Ming et al. [38] explained that these variations could be related to the fact that the biomass pyrolysis process is a mixture of parallel reactions. In this case, the *A* values were higher for blended SCG, indicating that it has a complex nature and its constituents follow a multi-phasic degradation reaction chemistry [36]. CH presented the lowest *A* values implying an easier thermal degradation process.

**Fig. 6:**
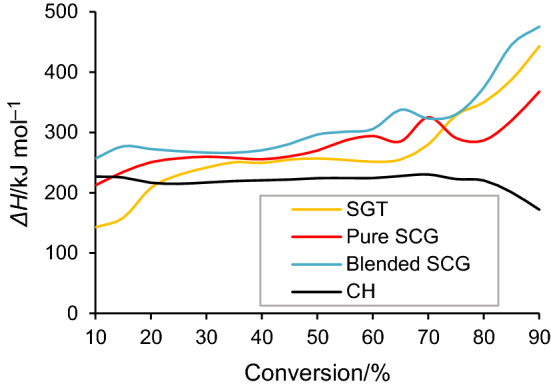
*ΔH* as a function of conversion for biomass samples at 10 °C min.^−1^

The *ΔH* values illustrate the energy consumed by the raw materials during the pyrolysis process for their conversion into the activated complex [11]. Based on Eq. (6) and the wide range obtained for *ΔH*, heating rate appears to have a negligible effect on the reaction enthalpy since the curves of *ΔH* vs α were practically superimposed for the four types of biomass waste. The positive *ΔH* values for the different biomass indicate that the pyrolysis reaction was endothermic (Fig. 6). For SGT, pure SCG and blended SCG, the *ΔH* increased from 10 to 90% conversion. However, for CH, *ΔH* increased slightly up to 75% conversion, before decreasing. This indicated a decay in the endothermicity of the CH biomass pyrolysis as conversion proceeded from 70 to 90%. A possible catalytic effect due to the high ash content of this sample could explain this behavior. By comparing *ΔH* and *E*_a_ values, small difference (~ 4–6 kJ mol^−1^) was noticed for the four types of lignocellulosic biomass, which implies beneficial conditions and feasible reactions for the production of the activated complex with the necessary *E*_a_. Moreover, this small difference affirms that the formation of products could be boosted by supplementing a low amount of energy (4 kJ mol^−1^) to the process [28, 36, 38]. In addition, the progress of the reaction could be related to the influence of the vapor pressure of the gaseous products released during the pyrolytic degradation. Therefore, the forward reaction is favored when the saturation vapor pressure of the primary pyrolysis products decreases.

**Fig. 7:**
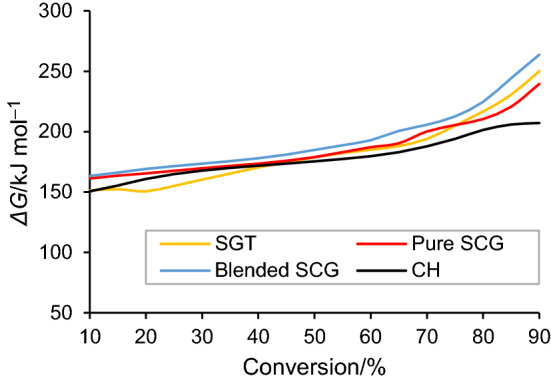
*ΔG* as a function of conversion for biomass samples at 10 °C min.^−1^

Gibbs free energy (*G*) represents the highest quantity of mechanical work that can be extracted from a specified portion of a selected substance. *ΔG* reveals the whole increase in the internal energy of the reaction system at the formation of the activated complex [54]. It also indicates the spontaneity of the system. The calculated *ΔG* for the tea and coffee wastes were all positive, indicating that the pyrolysis process was non-spontaneous. Therefore, an external energy has to be supplied to the pyrolysis process in order to promote thermal degradation reactions. Figure 7 represents the variation in *ΔG* with conversions at 10 °C min^−1^ for the four feedstocks. The *ΔG* values of SGT, pure SCG, blended SCG and CH were within the ranges 151–250 kJ mol^−1^, 161–239 kJ mol^−1^, 163–263 kJ mol^−1^ and 150–207 kJ mol^−1^, respectively. The *ΔG* values of SGT and coffee wastes increased slightly when the conversion increased from 10 to 65% followed by a significant increase in the *ΔG* values above 65%, which indicated that some of heat energy supplied to the pyrolysis system at high temperatures (> 350 °C) was surplus. These determined values were higher when compared with the *ΔG* values of castor bean residue (150.62–154.33 kJ mol^−1^) [28], exhausted coffee residue (145.1–156.6 kJ mol^−1^) [27] and banana leaves (39–74 kJ mol^−1^) [30]. These findings highlight the bioenergy potential of tea and coffee wastes.

**Fig. 8:**
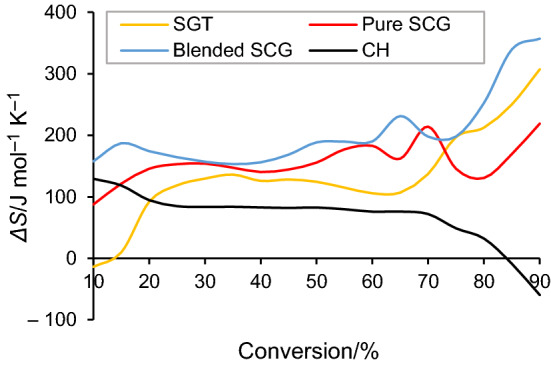
*ΔS* as a function of conversion for biomass samples at 10 °C min.^−1^

The entropy change (*ΔS*) is a state function indicating the randomness or degree of disorder of the reaction system [23]. The *ΔS* values fluctuated within the range of -14–307 J mol^−1^ K^−1^, 88–219 J mol^−1^ K^−1^, 158–357 J mol^−1^ K^−1^ and -60–129 J mol^−1^ K^−1^, for SGT, pure SCG, blended SCG and CH, respectively. Similar trends were observed for the *ΔS* for cattle manure (-71.2–316.2 J mol^−1^ K^−1^) [11] and rice (27.3–255.8 J mol^−1^ K^−1^) [38]. Therefore, both negative and positive values were evident for SGT and CH, whereas the *ΔS* values were entirely positive for two types of coffee ground waste. The negative *ΔS* values observed for SGT devolatilization during the first devolatilization stage (Fig. 8), which mainly involves the pyrolysis of extractives, imply that a slightly more ordered reaction system is initially obtained for this sample. This fact could explain the lowest energy requirement needed to carry out SGT pyrolysis at conversions lower than 20%, as previously mentioned. Generally, *ΔS* values increased when reaction conversion was increased from 10 to 90%, more evidently at conversions higher than 80%. Therefore, the formation of more disordered reaction systems occurred as the reaction progressed with an increase of the irreversible fraction, which leads to irrecoverable energy efficiency loss [14]. Exceptionally, the variation in *ΔS* for CH had the opposite behavior to that of the other samples above 80% conversion, where a considerable decline in the system entropy was observed down to negative values over 85% conversion, mainly involving lignin pyrolysis. In this case, the formation of a more ordered reaction system was achieved during the last devolatilization stage, which leads to reduce the energy efficiency loss. This finding could be explained by the highest ash content in CH (8.03%) compared to that of the other samples, acting as a volatiles cracking catalyst and promoting the formation of ordered carbon structures (char) instead of both gas and liquids. Similar findings have been also found for sorghum straw [53].

In summary, by combining the results obtained from the thermodynamic properties of the four samples, it can be deduced that the pyrolysis process of all types of biomass waste is endothermic. Therefore, a supplementary amount of energy needs to be added to the reactions system in order to boost the formation of products. The difference between the constituents of the four samples (extractives, hemicellulose, cellulose and lignin) and their properties leads to the variations in their reactivity during the pyrolysis process, which can be ranked in the following order of reactivity CH > SGT > pure SCG > blended SCG. The thermodynamic data obtained give an idea of the amount of energy needed to produce the conversion into the activated complex through the calculation of the *ΔH*, while the *ΔG* and *ΔS* at the activated complex provide information about the potential of the samples to reach this situation. These three terms have been related to the degree of conversion and properties of the solids in this work to allow taking informed decisions on the optimal extent of a targeted product. SGT, pure SCG and blended SCG samples have an important increase in the need of energy to reach the activation complex for conversions above 65% when the energy efficiency decreases. On the other hand, CH is the sample with the lowest need of energy keeping a similar energy efficiency through the whole conversion process. This is an important information to know the energy potential of the wastes that should be completed with the analysis of the overall standard enthalpy change related to the amount of final products to know the total energy available in a selected process.

## Conclusions

The physicochemical properties of SGT, pure SCG, blended SCG (with 50% torrefied barley), and CH were investigated. Moreover, the pyrolysis kinetics of the four types of biomass waste were studied using thermogravimetric data. Different isoconversional methods were used to determine the *E*_a_ profiles that significantly fluctuated between 150 and 500 kJ mol^−1^ in the 10–90% conversion range. A relationship was successfully established between the *E*_a_ obtained and the different biomass constituents (extractives, hemicellulose, cellulose and lignin) to exhibit four distinct stages, showing that while extractives decomposition was the least demanding reaction, lignin decomposition was the most demanding. The kinetic process was effectively reconstructed according to the obtained Friedman parameters. Additionally, the thermodynamic parameters were also evaluated. SGT, pure SCG and blended SCG samples have an important increase in the *ΔH* for conversions above 65% when the energy efficiency decreases. Of the different waste samples, CH was found to be the biomass waste with the highest reactivity (lowest average *E*_a_ 223 kJ mol^−1^), which could be related to its high hemicellulose and cellulose content, together with its high VM/FC ratio. This sample showed as well the lowest energy requirements (*ΔH*) while maintaining similar energy efficiency (*ΔS*) during the pyrolysis process compared to the other biomass wastes. All these findings could contribute to establishing potential industrial applications for tea and coffee wastes pyrolysis in order to generate bioenergy, high-value materials and chemicals.

## Supplementary Information

Below is the link to the electronic supplementary material.

Supplementary file1 (PDF 1016 KB)
