# Supplementary material for: Pyrolysis of tea and coffee wastes: effect of physicochemical properties on kinetic and thermodynamic characteristics
Source: J Therm Anal Calorim. 2023 Feb 10;148(6):2501–15. doi: 10.1007/s10973-022-11878-4 (PMC9911335; doi:10.1007/s10973-022-11878-4)
Supplement: Supplementary file 1 — Supplementary file1 (PDF 1016 KB) [file 10973_2022_11878_MOESM1_ESM.pdf]

Supplementary Information

Pyrolysis of tea and coffee wastes: Effect of physicochemical properties on kinetic and thermodynamic characteristics

Asma Ben Abdallah [1,2] · Aïda Ben Hassen Trabelsi [2] · María Victoria Navarro [3,*] · Alberto Veses [3] · Tomás García[3] · Daoued Mihoubi [2]

[1] Department of Energy Engineering, National Engineering School of Monastir, University of Monastir, Monastir 5000, Tunisia

[2] Laboratory of Wind Energy Management and Waste Energy Recovery (LMEEVED), Research and Technology Center of Energy (CRTEn), B.P. 95, 2050, Hammam-Lif, Tunisia

[3] Instituto de Carboquímica (ICB-CSIC), C/ Miguel Luesma Castán, 50018 Zaragoza, Spain

***** Corresponding author: navarro@icb.csic.es

Table S1 List of the different isoconversional methods used in the current study.

Methods		Expressions	Approximations used	Comments
Integral	KAS[1,2]	$\ln\left(\frac{\beta}{T^2}\right) = \ln\left(\frac{R.A}{E_a g(\alpha)}\right) - \frac{E_a}{RT}$	Murray and White approximation [3]: $p(x) \cong \frac{\exp(-x)}{x^2}$	The *Ea* is calculated from the slope of the plot $\ln(\frac{\beta}{T^2})$ vs. T^{-1}, for each α.
	FWO [4,5]	$\ln(\beta) = \ln\left(\frac{A.E_a}{R.g(\alpha)}\right) - 1.0518\frac{E_a}{RT} - 5.33$	Doyle approximation [6]: $p(x) \cong \exp(-2.315 + 0.4567x)$	The plot of $\ln(\beta)$ vs. T^{-1}, for each α gives a slope from which *Ea* is estimated
	Starink [7]	$\ln\left(\frac{\beta}{T^{1.8}}\right) = C_s - 1.0037\frac{E_a}{RT}$	Similarly to KAS and FWO methods	By plotting $\ln(\frac{\beta}{T^{1.8}})$ vs. T^{-1}, *Ea* can be determined from the slope
	Vyazovkin [8,9]	$\Phi(E_a) = \sum_{i=1}^{n}\sum_{j\neq i}^{n}\frac{I(E_a,T_{a,i})\beta_j}{I(E_a,T_{a,j})\beta_i}$	Senum-Yang approximation [10]: $I(E_a,T_a) = p(x) = a(\frac{b}{c})$ $a = \exp(-x)/x$ $b = x^7 + 70x^6 + 1886x^5 + 24920x^4 + 170136x^3 + 577584x^2 + 844560x + 357120$ $c = x^8 + 72x^7 + 2024x^6 + 28560x^5 + 21672x^4 + 880320x^3 + 1794240x^2 + 1572480x + 403200$	The value of apparent *Ea* for each α is obtained by minimizing $\Phi(E_a)$
Differential	Friedman [11]	$\ln[\beta_i.(\frac{d\alpha}{dT})_{\alpha,i}] = \ln(f(\alpha).A) - \frac{E_a}{RT}$	—	The plot of $\ln(\frac{d\alpha}{dt})$ vs. T^{-1} gives a slope from which Ea is evaluated

Table S2 Statistical treatment of different isoconversional methods at given conversion.

Biomass	R^2								Error/%	
	KAS		FWO		Starink		Friedman		Vyazovkin	
	Min	Max	Min	Max	Min	Max	Min	Max	Min	Max
SGT	0.99	1.00	0.99	1.00	0.99	1.00	0.98	1.00	0.00	1.30
pure SCG	0.97	1.00	0.97	1.00	0.97	1.00	0.96	1.00	0.10	2.83
blended SCG	0.96	1.00	0.96	1.00	0.96	1.00	0.94	1.00	0.01	3.87
CH	0.95	1.00	0.96	1.00	0.95	1.00	0.93	1.00	0.08	4.85

Table S3 Experimental-simulation fit quality at different heating rates.

Biomass wastes	β/°C min^{-1}	Fitting between experimental and simulated data		
		MAE/min^{-1}	RMSE/min^{-1}	R^2
SGT	10	0.17	0.19	0.99
	25	0.67	0.76	0.96
	50	1.37	1.70	0.96
	100	2.35	3.14	0.98
pure SCG	10	0.23	0.25	1.00
	25	0.73	0.85	0.99
	50	2.02	2.49	1.00
	100	4.15	4.70	1.00
blended SCG	10	0.48	0.55	1.00
	25	1.77	2.08	1.00
	50	4.35	5.09	1.00
	100	9.83	11.49	0.99
CH	10	0.11	0.14	1.00
	25	0.72	0.84	1.00
	50	2.35	2.79	1.00
	100	2.54	3.01	1.00

Table S4 Pre-exponential factors, A/s^{-1}, using the activation energy from Friedman method.

α	SGT	pure SCG	blended SCG	CH
10	$5,4·10^{12}$	$1,1·10^{18}$	$5,4·10^{21}$	$1,2·10^{20}$
15	$9,3·10^{13}$	$6,8·10^{19}$	$1,9·10^{23}$	$4,6·10^{19}$
20	$1,9·10^{18}$	$1,3·10^{21}$	$4,0·10^{22}$	$2,7·10^{18}$
25	$4,8·10^{19}$	$3,2·10^{21}$	$1,2·10^{22}$	$8,4·10^{17}$
30	$1,9·10^{20}$	$3,6·10^{21}$	$5,3·10^{21}$	$7,3·10^{17}$
35	$4,2·10^{20}$	$1.6·10^{21}$	$3,3·10^{21}$	$7,7·10^{17}$
40	$1,2·10^{20}$	$7,0·10^{20}$	$4,8·10^{21}$	$6,9·10^{17}$
45	$1,7·10^{20}$	$1,2·10^{21}$	$2,2·10^{22}$	$6,3·10^{17}$
50	$1,1·10^{20}$	$4,6·10^{21}$	$2,4·10^{23}$	$6,9·10^{17}$
55	$3,5·10^{19}$	$6,1·10^{22}$	$2,8·10^{23}$	$4,9·10^{17}$
60	$1,2·10^{19}$	$1,2·10^{23}$	$3,0·10^{23}$	$3,1·10^{17}$
65	$1,4·10^{19}$	$1,0·10^{22}$	$4,3·10^{25}$	$3,2·10^{17}$
70	$5,4·10^{20}$	$5,4·10^{24}$	$8,0·10^{23}$	$2,0·10^{17}$
75	$7,4·10^{23}$	$1,5·10^{21}$	$8,3·10^{23}$	$1,3·10^{16}$
80	$4,8·10^{24}$	$2,5·10^{20}$	$6,0·10^{26}$	$1,8·10^{15}$
85	$4,6·10^{26}$	$2,8·10^{22}$	$2,2·10^{31}$	$1,4·10^{13}$
90	$4,7·10^{29}$	$1,1·10^{25}$	$1,9·10^{32}$	$3,2·10^{10}$

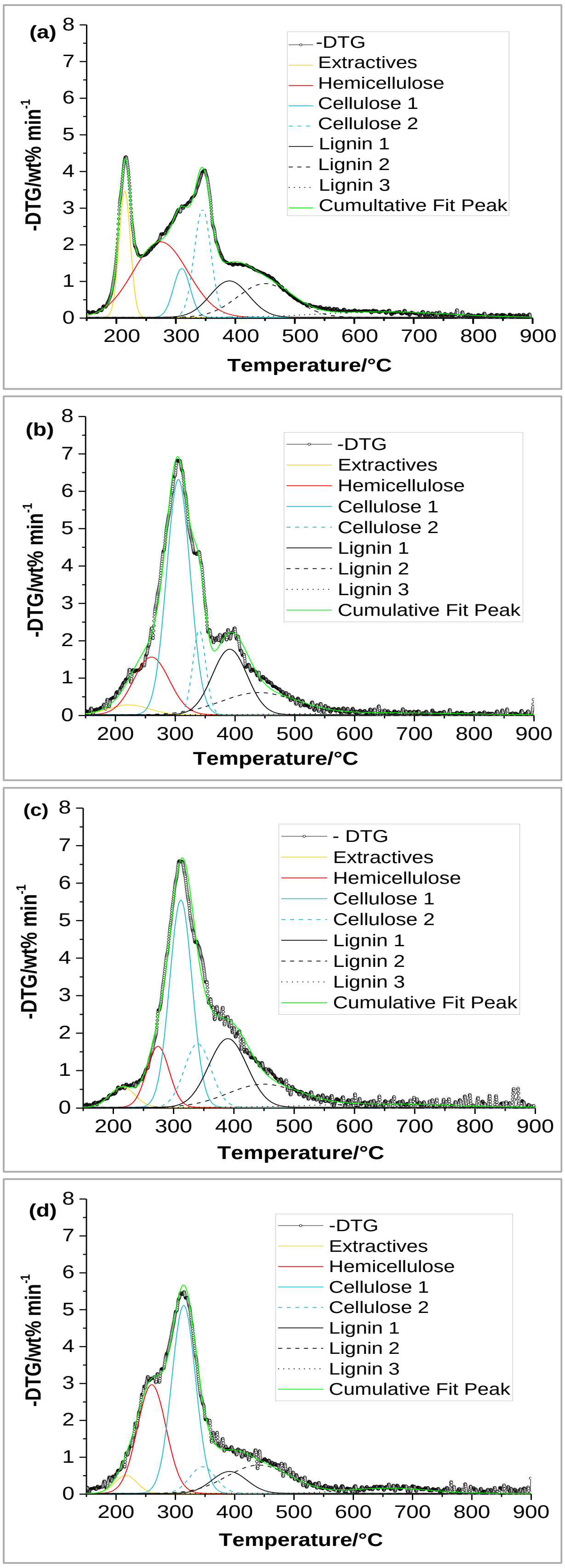

Fig. S1 Deconvolution of DTG curves of (a) SGT, (b) pure SCG, (c) blended SCG and (d) CH at 10 °C min^{-1}

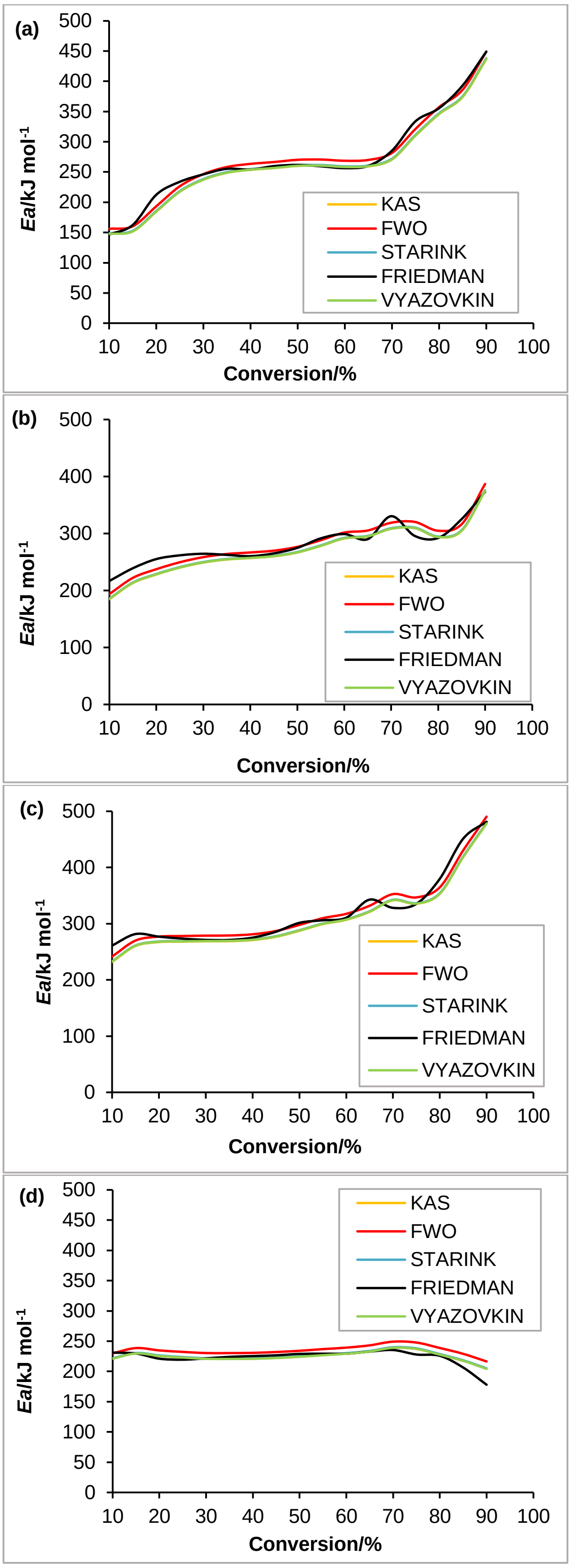

Fig. S2 *Ea* as a function of conversion for (a) SGT, (b) pure SCG, (c) blended SCG and (d) CH from different isoconversional methods

References

1. Kissinger HE. Reaction kinetics in differential thermal analysis. Anal Chem. 1957;29:1702–6.

2. Akahira T, Sunose T. Joint convention of four electrical institutes. Sci Technol. 1971;16:22–31.

3. Murray P WJ. Kinetics of the thermal dehydration of clays. Part IV. Interpretation of the differential thermal analysis of the clay mineral. Trans Br Ceram Soc. 1955;54:204–38.

4. Flynn JH, Wall LA. A quick, direct method for the determination of activation energy from thermogravimetric data. J Polym Sci Part B Polym Lett. 1966;4:323–8.

5. Ozawa T. A new method of analyzing thermogravimetric data. Bull Chem Soc Jpn. 1965;38:1881–6.

6. Doyle CD. Estimating isothermal life from thermogravimetric data. J Appl Polym Sci. 1962;6:639–42.

7. Starink MJ. The determination of activation energy from linear heating rate experiments: a comparison of the accuracy of isoconversion methods. Thermochim Acta. 2003;404:163–76.

8. Vyazovkin S. A Unified Approach to Nonisothermal Data. Unified Kinetic Processing. 1995;95–101.

9. Vyazovkin S, Wight CA. Model-free and model-fitting approaches to kinetic analysis of isothermal and nonisothermal data. Thermochimica Acta. 1999;340–341:53–68.

10. Senum GI, Yang RT. Rational approximations of the integral of the Arrhenius function. J Thermal Anal. 1977.

11. Friedman HL. Kinetics of thermal degradation of char-forming plastics from thermogravimetry. Application to a phenolic plastic. J Polym Sci Part C Polym Symp. 2007;6:183–95.
